# Assessment of tumor burden and response to therapy in patients with colorectal cancer using a quantitative ctDNA test for methylated *BCAT1/IKZF1*

**DOI:** 10.1002/1878-0261.13178

**Published:** 2022-01-24

**Authors:** Erin L. Symonds, Susanne K. Pedersen, Bernita Yeo, Hiba Al Naji, Susan E. Byrne, Amitesh Roy, Graeme P. Young

**Affiliations:** ^1^ 375963 Bowel Health Service Flinders Medical Centre Bedford Park Australia; ^2^ Cancer Research Flinders Health and Medical Research Institute Flinders University Bedford Park Australia; ^3^ Clinical Genomics Pty Ltd North Ryde Australia; ^4^ Department of Medicine College of Medicine and Public Health Flinders University Bedford Park Australia; ^5^ 375963 Department of Oncology Flinders Medical Centre Bedford Park Australia

**Keywords:** BCAT1, circulating tumor DNA, colorectal cancer, efficacy, IKZF1, methylation

## Abstract

Failure of colorectal cancer (CRC) treatment is due to residual disease, and its timely identification is critical for patient survival. Detecting CRC‐associated mutations in patient circulating cell‐free DNA is confounded by tumor mutation heterogeneity, requiring primary tumor sequencing to identify relevant mutations. In this study, we assessed *BCAT1* and *IKZF1* methylation levels to quantify circulating tumor DNA (ctDNA) and investigated whether this method can be used to assess tumor burden and efficacy of therapy. In 175 patients with CRC who were ctDNA‐positive pretreatment, ctDNA levels were higher with advancing stage (*P* < 0.05) and correlated with tumor diameter (*r* = 0.35, *P* < 0.001) and volume (*r* = 0.58, *P* < 0.01). After completion of treatment (median of 70 days [IQR 49‐109] after surgery, +/− radiotherapy, +/− chemotherapy), ctDNA levels were reduced in 98% (47/48) and were undetectable in 88% (42/48) of patients tested. For those with incomplete adjuvant chemotherapy after surgery, roughly half remained ctDNA‐positive (11/21, 52.4%). The presence of ctDNA after treatment was associated with disease progression (HR 9.7, 95%CI 2.5‐37.6) compared to no ctDNA. Assaying blood for ctDNA methylated in *BCAT1/IKZF1* has the potential for identifying residual disease due to treatment failure, informing a potential need for therapy adjustment in advanced disease.

## Graphical abstract

This study shows that the levels of circulating DNA methylated in *BCAT1* and/or *IKZF1* (ctDNA) correlate with colorectal tumor burden and inform response to treatment. This ctDNA methylation test aids detection of residual disease and may guide clinical decisions regarding additional therapy for patients with colorectal cancer.

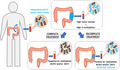

**Abbreviations**
CEA: carcinoembryonic antigen
cfDNA: cell‐free DNA
CI: confidence interval
CRC: colorectal cancer
CT: computed tomography
ctDNA: circulating tumor DNA
HR: hazard ratio
IQR: interquartile range
MRI: magnetic resonance imaging

## 1 Introduction

Survival from colorectal cancer (CRC) is dependent on the stage and treatment at diagnosis followed by careful monitoring of cases and effective therapy for recurrence. Up to 40% of CRC patients suffer clinically apparent recurrence following initial primary curative‐intent treatment [1]. Detection of residual disease and effective assessment of response to treatment are crucial for providing appropriate intervention to support mortality reduction.

Radiological imaging, usually computed tomography (CT), is used for evaluation of response to treatment and to monitor cases for recurrence. However, CT may not necessarily detect the actual tumor and the tumor mass must be sufficiently large, often > 1 cm, to be radiologically evident [2]. Furthermore, in the context of the increasing number of long‐term cancer survivors due to new anticancer therapies, repeated radiological assessments increase the radiation burden for the patient [3]. While blood testing for levels of carcinoembryonic antigen (CEA) is standard clinical practice for postresection monitoring of CRC patients, CEA has limited value due to poor sensitivity for residual disease and non‐neoplastic factors (such as smoking) adversely affecting assay specificity [4, 5, 6, 7].

There is a clinical need for highly sensitive and specific noninvasive tests to address the limitations of existing means for assessment of tumor response to therapy and detection of residual disease. Such methods would guide decision making with respect to the value of continuing a specific therapy in advanced disease, improve efficacy of initial therapy, and facilitate detection of clinical recurrence in early‐stage disease when initial therapy fails. Multiple studies have explored the value of assaying for circulating tumor DNA (ctDNA) as a means for detection of residual disease and recurrence and showed that this approach has the potential to salvage the chance of cure and to determine efficacy of therapy [8, 9, 10, 11, 12].

Detection of ctDNA in patients with CRC has primarily been based on assaying circulating cell‐free DNA (cfDNA) for somatic mutations associated with the development of CRC. This approach is confounded by the heterogeneity of tumor mutation profiles and that some approaches require sequencing of the primary tumor tissue to identify relevant mutations [9, 10, 11], which can come at a significant cost if comprehensive gene panels are used [13]. Recent research has demonstrated that detection of tumor‐specific differentially methylated regions improves assay sensitivity for ctDNA [7]. The enhanced sensitivity of ctDNA assays targeting methylated regions is because aberrant methylation can be detected in colorectal neoplastic tissue at the earliest stage and these epigenetic changes occur more commonly than the most frequently targeted mutational sequences associated with CRC [14, 15]. We have previously shown that for the methylated biomarkers *BCAT1* (branched chain amino acid transaminase 1) and *IKZF1* (IKAROS family zinc finger 1), 99% of CRC tissues have detectable methylation in either one or both genes, with 62% of the corresponding plasma samples having detectable levels of the methylated biomarkers [16].

Detection of circulating methylated *BCAT1* and *IKZF1* DNA shows promise for detection of residual disease after curative‐intent CRC surgery [16, 17]. The presence of methylated *BCAT1* and/or *IKZF1* ctDNA in blood during surveillance following curative‐intent treatment is indicative for disease recurrence and more sensitive than CEA testing [18, 19, 20]. Thus, changes in levels of these methylated biomarkers might dynamically reflect response to different therapies for CRC, such as surgery alone or surgery with either adjuvant or neoadjuvant therapy.

The primary aims of this study were to investigate whether quantitation of ctDNA levels by measuring the amount of methylated *BCAT1* and *IKZF1* DNA using a real‐time PCR assay reflects burden of CRC at diagnosis and whether changes in the amount of methylated *BCAT1/IKZF1* in blood accurately reflect response to different types of treatment.

## 2 Methods

### 2.1 Overview

For this observational study, the study cohort was retrospectively drawn from cases diagnosed with CRC undergoing prospective monitoring for recurrence. Cases were monitored according to applicable guidelines and blood samples were collected before treatment and as part of post‐treatment monitoring. Cases with detectable methylated *BCAT1* and/or *IKZF1* in blood (ctDNA‐positive) prior to initial treatment were included in the analysis. Levels of methylated ctDNA were compared with tumor burden, and changes in ctDNA levels were related to treatment type and treatment completion status.

### 2.2 Study population and clinical procedures

The study population comprised adults (over 18 years) undergoing treatment for primary CRC (adenocarcinoma) at Flinders Medical Centre (SA, Australia) from September 2011 to March 2020. Monitoring after initial treatment comprised regular clinical assessment and CT scans, supplemented by additional imaging as needed [21]. Venous blood was collected before and after initial therapy (30–200 days after cessation of treatment) and analyzed for methylated *BCAT1* and *IKZF1* DNA.

Patient demographic details, histopathology, imaging, and treatment details were collected. For colon and upper to mid‐rectal cancers, TNM staging and AJCC stage (AJCC guidelines version 8 [22]) were confirmed through clinicopathological findings at surgery. For those with low rectal tumors, staging was based on pretreatment staging MRI if neoadjuvant therapy was given. If synchronous cancers were present, designated stage was defined as the most advanced lesion.

Cases without invasive CRC (i.e., stage 0), inadequate staging, or failure to meet blood sampling requirements were excluded. Those who did not have blood collected within 200 days after ending initial treatment, or who died prior to treatment, were excluded from the post‐treatment analyses.

The study conformed to the standards set by the Declaration of Helsinki, was approved by the Southern Adelaide Clinical Human Research Ethics Committee (#134.045), and was registered at Australian and New Zealand Clinical Trials Registry (#12611000318987). The study was undertaken with the understanding and the written informed consent of all individuals.

### 2.3 Tumor burden assessment

Tumor burden at diagnosis was considered by stage, size, and volume. Size was considered as the maximum diameter of the primary tumor, as well as the sum of all maximum diameters when synchronous tumors or metastatic lesions were present as per WHO guidelines [23]. For individuals who had undergone surgery and where more than one size dimension was recorded, tumor volume of the primary lesion was calculated with the modified ellipsoid formula (4/3π(longest diameter)^2^*(width)) [24].

### 2.4 ctDNA quantification

Blood was collected in K3‐EDTA blood tubes and processed to plasma within 4 h by two consecutive centrifugation steps (1500 ***g***, 10 min). cfDNA was extracted from 3.9–4.5 mL plasma, bisulphite converted and a multiplexed real‐time quantitative PCR assay was used to determine the amount of methylated *BCAT1*/*IKZF1* DNA and total amount of DNA (*ACTB)* as previously described [19]. All samples were analyzed in triplicate and mass was estimated using PCR cycle threshold (Ct) values derived from a 2.5‐fold serial dilution of known mass inputs. Total methylated DNA was expressed as a percentage of the mass of total cfDNA in plasma (hereinafter referred to as “%methylation”). Detection of methylation in either *BCAT1* or *IKZF1* in a sample was reported as ctDNA‐positive.

### 2.5 ctDNA levels with treatment

Changes in ctDNA levels between pre‐ and post‐treatment samples were assessed relative to different treatment types and according to treatment completion status. This included those where initial treatment was completed according to standard of care (“complete treatment”), namely surgery only (for early‐stage CRC), surgery with adjuvant chemotherapy (for those with advanced/metastatic CRC), and neoadjuvant therapy plus surgery (for those with locally advanced low rectal cancer). Analysis was also done for individuals who had not completed guideline appropriate treatment (“incomplete treatment”), for example, when the patient declined adjuvant chemotherapy or stopped therapy early due to side effects.

### 2.6 Statistical analyses

Descriptive variables and ctDNA levels were expressed as median with interquartile range (IQR). The Mann–Whitney test or the Kruskal–Wallis test were used to compare ctDNA levels between stages of disease as data were found to not have a normal distribution (D'Agostino–Pearson test). Pearson correlation analysis was used to determine the correlation between ctDNA levels and tumor burden estimates. Changes to ctDNA levels with treatment were assessed with paired Wilcoxon signed‐rank tests, and chi‐square tests were used to compare proportions of patients with detectable ctDNA after different treatments. Disease progression status (i.e., radiological confirmed spread or recurrence of cancer) post‐treatment was assessed over a minimum follow‐up time of 12 months. Kaplan–Meier curves and hazard ratio (HR) with 95% confidence intervals (CI) were calculated with Cox regression analysis and compared the disease progression for the post‐treatment ctDNA‐positive and ctDNA‐negative groups. Statistical analyses were performed with graphpad Prism (version 6.07, GraphPad Software, Inc., La Jolla, CA, USA) and STATA (v16.0, StataCorp LLC, TX, USA). *P* values less than 0.05 were considered statistically significant.

## 3 Results

### 3.1 Patient population

**Fig. 1:**
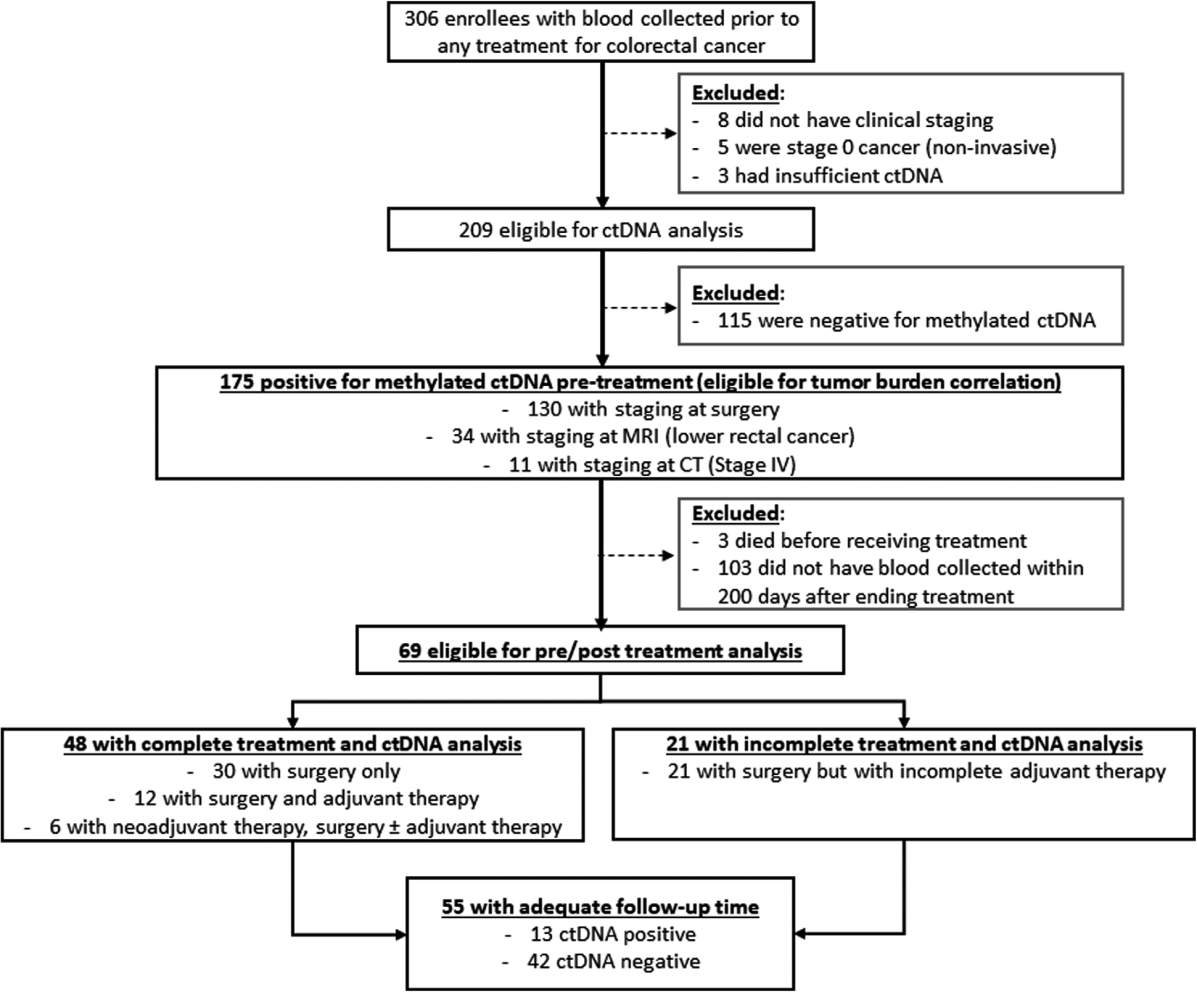
Disposition of cases and how they were selected for the main analyses. cfDNA: cell‐free DNA; ctDNA: circulating tumor DNA; MRI: magnetic resonance imaging; CT: computed tomography.

There were 306 CRC patients that had blood collected at diagnosis of CRC and prior to any treatment (189 males (61.4%); median age 68.8 years, range: 29.7–85.9 years). Following exclusions (Fig. 1), 290 (94.2%) patients had a ctDNA test result prior to primary treatment; 175 (60.9%) of these were positive for methylated *BCAT1/IKZF1* ctDNA. These included 19.4% (34/175) low rectal cancer staged at MRI and 6.3% (11/175) who were diagnosed as stage IV based on CT imaging and who did not have surgery. The remaining 130 patients had full staging at surgery. Table 1 shows the demographics and tumor characteristics of those eligible for the analyses.

**Table 1:** Characteristics of patients eligible for analyses. IQR, interquartile range. AJCC, American Joint Committee on Cancer.

Patient or tumor feature	Tumor burden correlation analysis (all cases, *N* = 175)	Pre/post treatment analysis (subset, *N* = 69)	*P* value^*^
Age, median y (IQR)	68.6 (58.5‐76.1)	68.7 (58.6‐76.3)	0.68
Age < 65 years, *N* (%)	66 (37.7)	25 (36.2)	0.83
Age ≥ 65 years, *N* (%)	109 (62.3)	44 (63.8)	
Gender, *N* (%)			
Female	63 (36.0)	23 (33.3)	0.69
Male	112 (64.0)	46 (66.7)	
Source of staging, *N* (%)			
Imaging only	45 (25.7)	9 (13.0)	0.03
Surgery and imaging	130 (74.3)	60 (87.0)	
T stage^a^, *N* (%)			
T1	1 (0.6)	1 (1.4)	0.39
T2	18 (11.0)	5 (7.3)	
T3	107 (65.2)	52 (75.4)	
T4	38 (23.2)	11 (15.9)	
*N* stage^a^, *N* (%)			
N0	73 (44.5)	34 (49.3)	0.49
N1	39 (23.8)	19 (27.5)	
N1c	8 (4.9)	4 (5.8)	
N2	44 (26.8)	12 (17.4)	
M stage, *N* (%)			
M0	136 (77.7)	66 (95.7)	<0.01
M1	39 (22.3)	3 (4.3)	
Location of distant metastases			
Metastases in liver^b^	31/39 (79.5%)	1/3 (33.3%)	
Metastases in lung^b^	15/39 (38.5%)	3/3 (100.0%)	
Metastases in other locations^b^	11/39 (28.2%)	1/3 (33.3%)	
Stage (AJCC)			
I	14 (8.0%)	6 (8.7%)	0.01
II	53 (30.3%)	27 (39.1%)	
III	69 (39.4%)	33 (47.8%)	
IV	39 (22.3%)	3 (4.4%)	
Location of primary tumor			
Proximal colon^c^	58 (33.1%)	26 (37.7%)	0.54
Distal colon	56 (32.0%)	24 (34.8%)	
Rectum	61 (34.9%)	19 (27.5%)	
Maximum diameter of primary, median mm (IQR) (*n* = 165; *n* = 69)	50 (36‐65)	45 (35‐60)	0.44

^a^ Not available for all individuals. ^b^Some individuals had more than one metastatic site. ^c^Proximal colon included sites proximal to the splenic flexure. *Comparison of subset (*n* = 69) to all cases (*n* = 175).

### 3.2 Association between ctDNA and tumor burden

The median level of methylated ctDNA prior to any treatment was 0.29% (IQR 0.07–2.49%). ctDNA levels correlated with features of tumor severity, with higher levels correlating with increasing T‐stage, M‐stage, and presence of extramural venous invasion (EMVI) (Table 2). The median ctDNA level was 7.8 fold higher for stage T4 compared to stage T2. When distant metastatic disease was present, the median ctDNA level was 42.5 fold higher than the levels measured in patients without metastatic disease (Table 2). A relationship was seen between overall AJCC stage and amount of ctDNA, with all stages having higher levels than stage I (Table 2). This was also observed when considering individuals’ levels of methylated ctDNA according to T stages stratified by overall stage (Fig. S1). The measured median levels of methylated ctDNA did not differ in patients with liver or lung metastatic lesions; however, the levels were significantly higher than those in patients with other single site metastatic lesions (Fig. S2).

**Table 2:** Relationship between tumor features and methylated ctDNA levels (“%methylated”) prior to any treatment.

Tumor feature or stage (*n* = 175)	%methylated ctDNA Median (IQR)	*P* value*
Location of primary tumor		
Proximal colon (*n* = 58)	0.22 (0.07–2.58)	0.97
Distal colon (*n* = 56)	0.33 (0.07–2.56)	
Rectum (*n* = 61)	0.36 (0.05–2.26)	
Differentiation^a^		
Moderate‐well (*n* = 96)	0.17 (0.06–1.45)	0.30
Poor (*n* = 31)	0.34 (0.04–4.44)	
Lymphatic invasion^a^		
No (*n* = 80)	0.23 (0.05–1.54)	0.41
Yes (*n* = 49)	0.18 (0.07–1.80)	
Extramural venous invasion^a^		
No (*n* = 100)	0.17 (0.05–1.49)	0.01
Yes (*n* = 28)	0.99 (0.14–4.83)	
T stage^b^		
T1 (*n* = 1)	0.04 (0.04–0.04)	<0.01^c^
T2 (*n* = 18)	0.05 (0.02–0.11)	
T3 (*n* = 107)	0.35 (0.08–1.91)	
T4 (*n* = 38)	0.39 (0.11–4.02)	
*N* stage^b^		
N0 (*n* = 73)	0.21 (0.05–1.12)	0.25
N1 (*n* = 39)	0.18 (0.08–1.91)	
N1c (*n* = 44)	0.55 (0.07–5.75)	
N2 (*n* = 8)	0.67 (0.11–4.46)	
M stage		
M0 (*n* = 136)	0.17 (0.06–0.95)	<0.01
M1 (*n* = 39)	7.22 (1.15–28.91)	
Overall stage		
I (*n* = 14)	0.05 (0.02–0.08)	<0.01
II (*n* = 53)	0.23 (0.09–1.12)	
III (*n* = 69)	0.17 (0.07–1.59)	
IV (*n* = 39)	7.22 (1.15–28.91)	

^a^ Pathology features were only able to be assessed in those who had undergone surgery prior to other therapies. ^b^Stage not available for all individuals. ^c^Stage T2 was significantly different to T3 and T4 (Stage T1 excluded from analysis due to small sample size).

* *P* value: Mann–Whitney test or Kruskal–Wallis test to compare median methylation levels within each tumor feature.

**Fig. 2:**
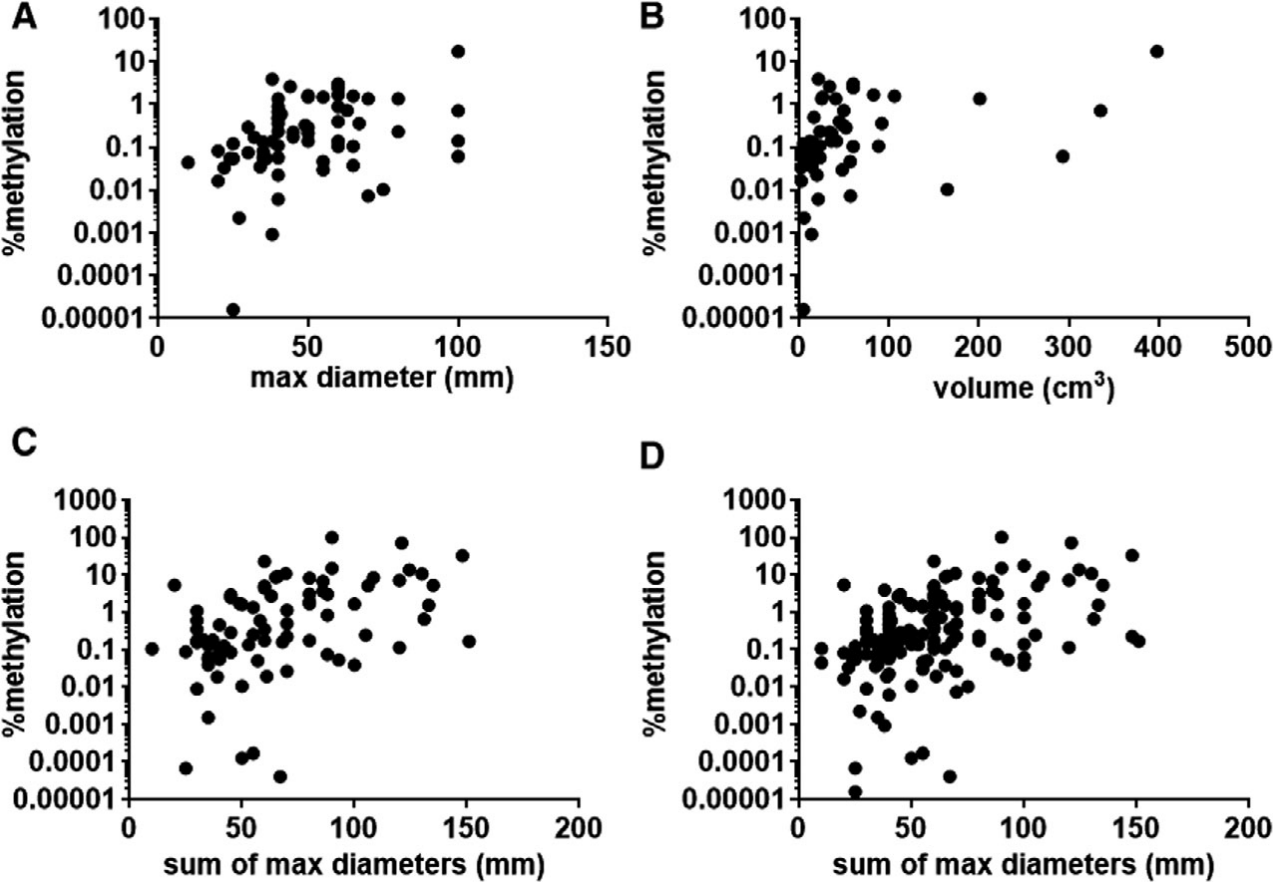
Relationship between the amount of methylated ctDNA in circulation and (A) maximum tumor diameter in patients with CRC stages I and II (*n* = 66, *r* = 0.359, *P* = 0.003), (B) estimated tumor volume in patients with CRC stages I and II (*n* = 50, *r* = 0.586, *P* < 0.001), (C) sum of maximum tumor diameters (primary and evident metastatic sites) in patients with CRC stages III and IV (*n* = 85, *r* = 0.319, *P* = 0.003), and (D) sum of maximum tumor diameters (primary and evident metastatic sites) in all patients (*n* = 152, *r* = 0.321, *P* < 0.001). Statistical correlations were performed with Pearson correlation analysis.

To assess the relationship between tumor size with ctDNA levels, stage I and II CRC were firstly assessed separately to limit the tumor mass to the primary site in the colorectum. A significant correlation was found between ctDNA levels and maximum diameter (*r* = 0.36, *P* < 0.01; Fig. 2A), as well as estimated tumor volume (*r* = 0.59, *P* < 0.01; Fig. 2B). For advanced CRC, total tumor burden (when expressed as the sum of maximum diameters for the primary and each evident metastatic lesion) and ctDNA levels were correlated (*r* = 0.32, *P* < 0.01; Fig. 2C). A correlation was also observed when considering all stages of CRC (*r* = 0.32, *P* < 0.01; Fig. 2D).

### 3.3 Treatment‐induced changes to ctDNA levels

**Fig. 3:**
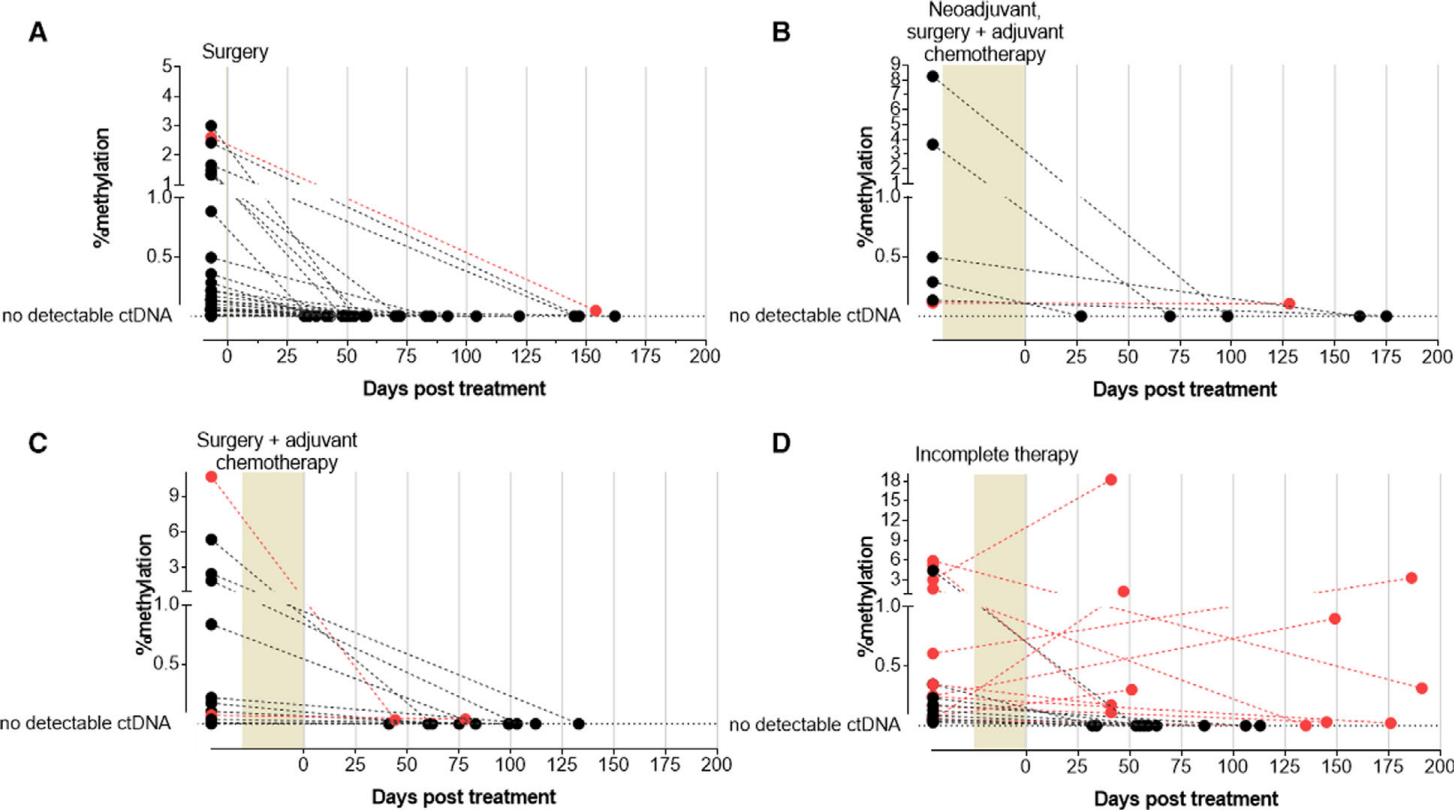
Levels of methylated *BCAT1/IKZF1* (% methylation—see Methods) in patients with CRC before and after cessation of treatment with (A) surgery (*n* = 30); (B) neoadjuvant therapy, surgery, and adjuvant chemotherapy (*n* = 6); (C) surgery and adjuvant chemotherapy (*n* = 12); and (D) incomplete adjuvant chemotherapy (*n* = 21). Each marker with the joining line represents the methylation levels before and after treatment. The pink markers indicate patients that had detectable ctDNA levels pre‐ and post‐treatment.

Forty‐eight patients had blood collected before (median 7.0 days; IQR 5.0–17.0) and after (70.5 days; IQR 50.0–102.0) complete treatment (Fig. 1). This subset included 30 with stage I or II disease that had surgery alone, 6 with lower rectal cancers that had neoadjuvant therapy followed by surgery (with or without adjuvant chemotherapy), and 12 with regional or metastatic disease that had surgery (primary and metastasectomy) followed by a complete course of adjuvant therapy). Treatment resulted in a decrease in ctDNA levels in 97.9% (47/48) of individuals relative to the pretreatment level (*P* < 0.05), with no detectable ctDNA present in 87.5% (42/48) of individuals after treatment (Fig. 3A,B,C, and Table 3).

**Table 3:** ctDNA test results following different treatments and treatment status.

Treatment	*N*	%methylation in positive cases		Post‐treatment ctDNA test results	
		Pretreatment (median, IQR)	Post‐treatment (median, IQR)^a^	No. with decreased ctDNA levels compared to pretreatment, *n* (%)	No. with no detectable ctDNA, *n* (%)
All cases	69	0.18 (0.06–1.35)	0.11 (0.03–0.61)	63 (91.3)	52 (75.4)
Surgery alone	30	0.14 (0.05–1.00)	0.00 (0.00–0.05)*	29 (96.7)	27 (90.0)
Complete surgery and adjuvant chemotherapy	12	0.20 (0.05–2.34)	0.04 (0.03–0.04)	12 (100)	10 (83.3)
Complete neoadjuvant therapy plus surgery (+/− further chemotherapy)	6	0.39 (0.13–4.82)	0.11	6 (100)	5 (83.3)
Surgery, but incomplete or no adjuvant chemotherapy	21	0.23 (0.07–1.15)	0.30 (0.03–1.28)	16 (76.2)	10 (47.6)

* *P* = 0.02 compared to pretreatment ctDNA (circulating tumor DNA) levels. ^a^Median post‐treatment was calculated for the cases that remained positive post‐treatment, including *n* = 3 after surgery, *n* = 2 after complete surgery and adjuvant chemotherapy, *n* = 1 after neoadjuvant therapy, *n* = 11 after incomplete treatment.

Blood samples were collected from 21 patients with incomplete treatment. All patients underwent surgery of the primary lesion, but either had no adjuvant treatment or stopped adjuvant treatment early. Blood was collected 6.0 days (IQR 5.0–33.5) prior to treatment, and 59.0 days (44.0–140.0) after treatment stopped. Blood collection and demographic details were similar to that for the group that had completed planned therapy with surgery plus adjuvant chemotherapy (Table S1). It was observed that 52.4% (11/21) remained ctDNA‐positive post‐treatment, with no significant difference in the median ctDNA level from pretreatment (Table 3; Fig. 3D, *P* = 0.20).

### 3.4 Association between a positive ctDNA result post‐treatment and disease progression

Of all patients with a post‐treatment blood sample, 17 of the 69 patients had detectable ctDNA—with a significantly lower proportion of positive ctDNA results observed following complete treatment (6/48; 12.5%) compared to following incomplete treatment (11/21; 52.4%).

**Fig. 4:**
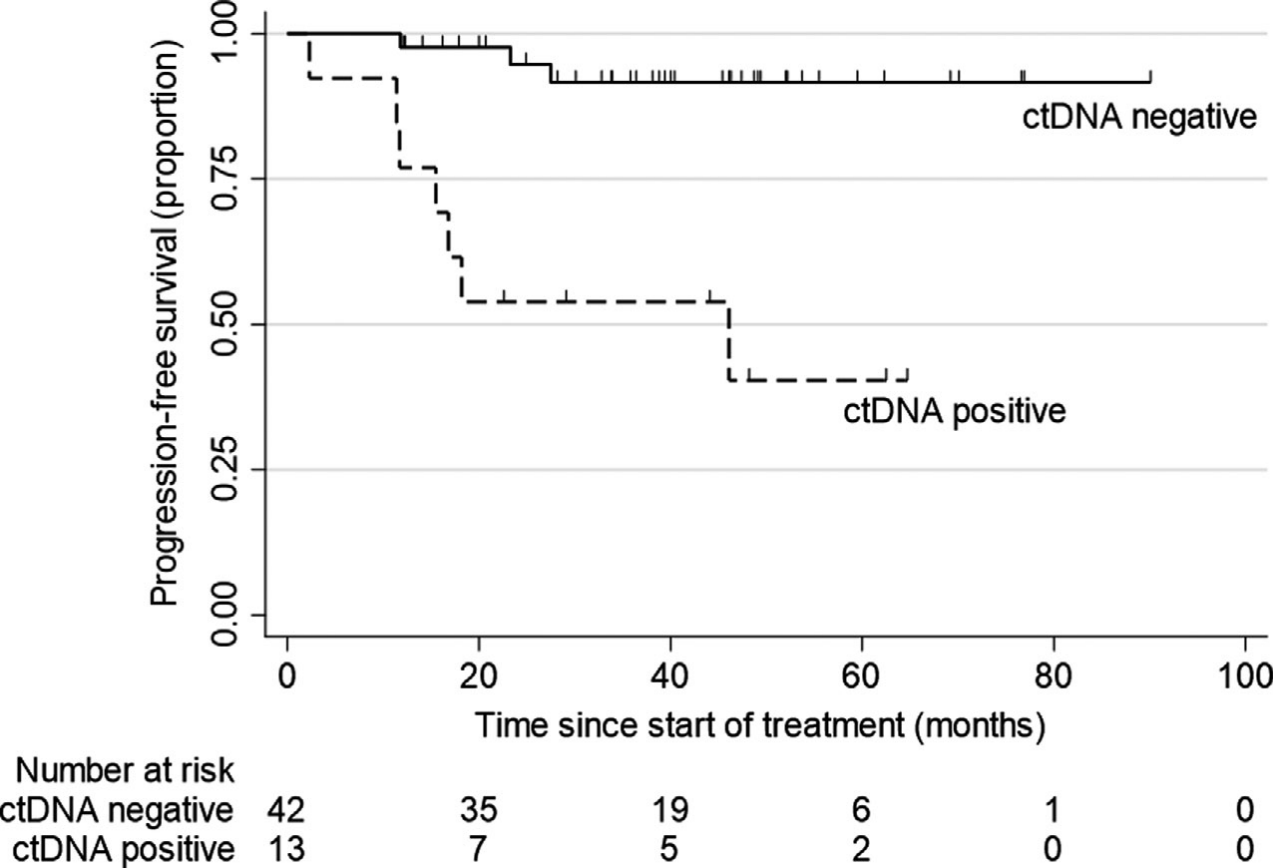
Kaplan–Meier curves for progression‐free survival stratified according to post‐treatment detection of ctDNA (methylated *BCAT1/IKZF1*); positive ctDNA, *n* = 13, negative ctDNA, *n* = 42. Hazard ratio for disease progression for those with a post‐treatment positive ctDNA was 9.69, 95% confidence interval 2.50‐37.59. Statistical analysis was with Cox regression analysis.

Of the 69 cases included for primary analysis, there were 55 cases with adequate follow‐up time (37.9 months, IQR 20.5–49.2) who were reviewed for disease recurrence or progression, including 42 with a negative ctDNA post‐treatment result (3 stage I, 20 stage II, 19 stage III) and 13 with a positive ctDNA result (1 stage II, 9 stage III and 3 stage IV). There were significant differences in disease recurrence/progression status between the two groups, with the post‐treatment positive ctDNA group having a HR of 9.7 (95% confidence interval 2.5–37.6) for disease recurrence/progression (Fig. 4). Of the 42 cases that were ctDNA‐negative post‐treatment, 7.1% (*n* = 3) had disease recurrence or progression, compared to 53.8% of the cases who were found to be ctDNA‐positive post‐treatment (*n* = 7/13). Of all 13 cases with a positive ctDNA result, the %methylation was significantly higher in the 7 individuals with confirmed disease progression or recurrence (%methylation 0.32%, IQR 0.17‐3.33%) compared to the 6 with no evidence of disease (median %methylation 0.04%, IQR 0.03‐0.30; *P* = 0.01).

## 4 Discussion

This observational study showed a correlation between the levels of methylated *BCAT1/IKZF1* ctDNA and tumor burden. Furthermore, methylation levels responded to the range of treatments applicable to CRC of different stage and location. ctDNA was detected in 52% of the cases following incomplete treatment, and an association between persisting ctDNA and progressing or recurrent disease was observed for almost two‐thirds of these cases. Thus, measuring ctDNA based on levels of methylated *BCAT1/IKZF1* informs response to the therapy for CRC.

Imaging with CT is used clinically to assess tumor burden, but there are significant limitations with this methodology, given the poor sensitivity for smaller lesions and cumulative radiation exposure. The use of blood‐based biomarkers, such as ctDNA, as surrogate markers for tumor burden, provides new methods for surveillance and monitoring of CRC [9]. Most studies have focused on tumor mutation biomarkers for detection of ctDNA, but there are challenges in identifying individual‐specific mutations [25] as these differ between patients [26, 27], and the mutation profile can change with disease progression [28]. Conversely, ctDNA detection based on DNA methylation biomarkers shows promise with the potential to be a widely applicable biomarker for CRC as the methylation profiles are not affected by tumor heterogeneity or stage. In the current study, we have extended on our previous findings [16, 29, 30] and have shown a close relationship between the amount of circulating methylated *BCAT1/IKZF1* DNA and tumor burden at diagnosis. With regard to stage, levels rose with deeper invasion (T stage) and development of distant metastases and EMVI. These relationships are consistent with earlier findings using a qualitative assay [29, 30]. Levels of methylated *BCAT1/IKZF1* DNA at the time of diagnosis could serve as a baseline to judge the significance of altered levels in repeated measures as treatment is implemented, adjusted, and monitored.

A range of variables, in addition to technological factors such as analytical sensitivity, determine whether tumor‐derived DNA is present and detectable in the circulation. It is known that tumor growth dynamics, ctDNA half‐life, and rate of shedding into the circulation can all strongly influence ctDNA levels. Abundance of ctDNA has been observed to be low for small mass or early‐stage cancers and this poses technological challenges [8] as well as creating uncertainty for the best timing for sampling blood. The relationship between levels of ctDNA and cancer stage as observed here and confirmed by others [9, 10] is consistent with the model that as cancers invade, the number of ctDNA molecules increases due to egress into the circulation [8].

To further examine the relationship between levels of methylated ctDNA and tumor burden, we compared ctDNA levels with different measures of tumor mass based on size and volume. The standard clinical tool to evaluate response of radiologically visible disease (usually metastatic CRC) to treatment is based on RECIST (Response Evaluation Criteria in Solid Tumors) [31] which uses imaging to assess changes in the longest axial diameter of tumors (typically just a single distant metastasis). However, these size measurements do not necessarily reflect tumor burden [32, 33]. By correlating methylation levels with multiple measures of mass, we found that ctDNA levels were consistently increased with greater tumor mass. This observation is consistent with other studies [32, 34].

We then tested the ability of this quantitative ctDNA assay to reflect treatment‐induced reductions in tumor burden in the 69 cases where a subsequent blood test result was available following cessation of therapy. In the 48 cases with complete treatment, the levels of methylated *BCAT1* and *IKZF1* DNA dropped substantially, with the majority (87.5%) of the patients having undetectable levels. In the 6 that remained positive for ctDNA after completing recommended treatment, the levels were very low. This trend was observed regardless of treatment type. These data can be used to inform clinical decisions regarding additional therapy versus active surveillance and perhaps reduce patient anxiety when making these types of decisions.

The biggest challenge for prognosis is whether ctDNA can indicate the presence of residual disease. Residual disease is a difficult condition to verify without long‐term follow‐up via usual surveillance methods. In addition, efforts to improve sensitivity of blood tests where ctDNA abundance in plasma is low may create issues of specificity [8], which poses challenges if the test result is to be used to initiate chemotherapy without radiological confirmation of residual tumor. However, the presence of residual disease when a ctDNA biomarker is positive is supported by studies that have demonstrated a relationship between ctDNA and recurrence [7, 11, 18, 19, 20]. To improve understanding of ctDNA in relation to residual disease in the current study, we assessed the changes in ctDNA levels in patients where treatment was known to be incomplete. It was found that more than half (11 out of 21) remained positive and 5 of the 11 had higher ctDNA levels compared to the levels measured prior to treatment. While the study was not designed to include survival as a primary outcome, and because ctDNA detection might reflect residual disease, we compared disease progression‐free survival in ctDNA‐positive and ctDNA‐negative cases. Those who were ctDNA‐positive had a significantly shorter time without disease progression (HR 9.7), consistent with detection of residual disease by this ctDNA test. Given that circulating levels of methylated *BCAT1/IKZF1* DNA respond to treatment, additional studies will be needed to show if quantitation improves capacity to salvage chance of cure by tailoring therapy to the individual case and especially to adjust therapy to deal with disease not otherwise able to be identified.

This study was designed to test the potential of this quantitative ctDNA assay to assess tumor mass and to indicate the presence of residual cancer without the need to personalize the ctDNA blood test based on tissue DNA profiling. In contrast to NGS‐based ctDNA tests which are resource‐intensive, the methylated *BCAT1/IKZF1* qPCR‐based ctDNA test is low cost and turnaround time is short [7, 12]. The strength, even though observational in the context of usual care for cases where sampling was dependent on clinical opportunity and focused on pre‐ and post‐treatment levels, was that we could observe responses across the full spectrum of therapeutic options for colon and rectal cancers. Studies are now needed to determine the best timing of sampling since sampling was opportunistic with blood collected at varying times pre‐ and post‐treatment. Investigation of methylated ctDNA changes in patients undergoing serial longitudinal plasma sampling during monitoring is also needed to determine whether the actual post‐treatment level of methylated *BCAT1/IKZF1* is an indicator of minimal residual disease and survival.

## 5 Conclusions

This quantitative ctDNA test measuring levels of methylated *BCAT1/IKZF1* DNA in blood reflects tumor burden at diagnosis and provides a baseline for demonstrating response to differing therapeutic approaches for colon and rectal cancer. A positive ctDNA result post‐treatment was associated with disease progression. This test therefore has the potential to identify treatment failure due to residual disease and inform adjustment of therapy to improve chances of survival. In addition, it promises capacity to assess and identify changes in tumor burden that could guide decisions about the value of continuing a specific therapy in advanced disease.

## Conflict of interest

GPY is a paid consultant of Clinical Genomics. SKP is a paid employee of Clinical Genomics. Other authors have no conflicts of interest to declare.

### Peer review

The peer review history for this article is available at https://publons.com/publon/10.1002/1878‐0261.13178.

## Author contributions

ELS and GPY conceived the study and analysis with input from SHP and AR. SEB and HAN collected the data. AR and SEB provided clinical review of cases. ELS, SKP, BY, AR, and GPY audited the data. ELS conducted the statistical analysis. ELS and GPY wrote the first draft of the manuscript. SKP, BY, HAN, SEB, and AR revised the manuscript.

## Supporting information


**Fig. S1.** Individual levels of ctDNA (expressed as the percentage of methylated *BCAT1/IKZF1* DNA measured in total cfDNA, “%methylation levels”) for patients according to T stages stratified by overall stage.
**Fig. S2.** Individual ctDNA levels (expressed as the percentage of methylated *BCAT1/IKZF1* DNA measured in total cfDNA, “%methylation levels”) for patients according to site of metastasis.
**Table S1.** Comparison of patients with and without complete treatment comprising surgery and adjuvant chemotherapy.

## Data Availability

Data may be made available on request to the corresponding author.
