# Supplementary material for: Assessment of tumor burden and response to therapy in patients with colorectal cancer using a quantitative ctDNA test for methylated BCAT1/IKZF1
Source: Mol Oncol. 2022 Jan 24;16(10):2031–41. doi: 10.1002/1878-0261.13178 (PMC9120880; doi:10.1002/1878-0261.13178)
Supplement: Supplementary file 1 — Fig. S1. Individual levels of ctDNA (expressed as the percentage of methylated BCAT1/IKZF1 DNA measured in total cfDNA, “%methylation levels”) for patients according to T stages stratified by overall stage. Fig. S2. Individual ctDNA levels (expressed as the percentage of methylated BCAT1/IKZF1 DNA measured in total cfDNA, “%methylation levels”) for patients according to site of metastasis. Table S1. Comparison of patients with and without complete treatment comprising surgery and adjuvant chemotherapy. [file MOL2-16-2031-s001.docx]

**Supplementary Figures**

**Supplementary Figure 1:** Individual levels of ctDNA (expressed as the percentage of methylated *BCAT1/IKZF1* DNA measured in total cfDNA, “%methylation levels”) for patients according to T stages stratified by overall stage. The solid bars show the median levels, and the markers represent each patient’s ctDNA level: closed squares- T1 stage; open circles- T2 stage; closed diamonds- T3 stage; open squares- T4 stage. T1- stage I, n=1; T2- stage I, n=13; T2- stage III, n=5; T3- stage II, n=41; T3- stage III, n=50; T3- stage IV, n=16; T4- stage II, n=12; T4- stage III, n=14; T4- stage IV, n=12.

**Supplementary Figure 2:** Individual ctDNA levels (expressed as the percentage of methylated *BCAT1/IKZF1* DNA measured in total cfDNA, “%methylation levels”) for patients according to site of metastasis. The solid bars show the median levels, and the markers represent each patient’s ctDNA level. Liver only, n=17; lung only, n=4; liver and lung, n=8; other single site, n=4; liver and other sites, n=6.

**Supplementary Table 1:** Comparison of patients with and without complete treatment comprising surgery and adjuvant chemotherapy.

|  | **Completed surgery and adjuvant treatment (n=12)** | **Surgery with incomplete adjuvant treatment (n=21)** | **P value** |
| --- | --- | --- | --- |
| **No. male (%)** | 7 (58.3%) | 16 (76.2%) | 0.28 |
| **Median age (IQR), y** | 74.0 (53.0-80.9) | 71.5 (61.3-77.9) | 0.93 |
| **Days between first blood and start of treatment (IQR)** | 6.0 (5.0-6.8) | 6.0 (5.0-33.5) | 0.79 |
| **Days between second blood and end of treatment (IQR)** | 80.5 (60.5-102.0) | 59.0 (44.0-140.0) | 0.76 |
| **Pre-treatment median %methylation (IQR)** | 0.18% (0.04-1.91%) | 0.23% (0.07-1.15%) | 0.67 |
| **Post-treatment no. positive (%)** | 2 (16.7%) | 11 (52.4%) | 0.04 |

IQR: interquartile range
